# Headache research - where are we heading?

**DOI:** 10.1186/1129-2377-14-S1-I3

**Published:** 2013-02-21

**Authors:** Rigmor Jensen

**Affiliations:** 1 Danish Headache Centre, Department of Neurology, Glostrup Hospital, Denmark

## 

Since the pionering work with the IHS-classification in 1988 and the triptan era in the early nineties, the field of headache research has expanded exponentially. Slowly but steadily headache has been moved from a trivial and neglected disorder to be respected and acknowledged as illustrated by the nomination of 2012 as The Year of Headache in The International Association of Pain. Almost all areas of headache research are now flourishing from genetics, experimental animal and human research, epidemiological and costs studies to new treatments and optimized care.

Despite this scientific explosion, why are millions of headache patients still suffering from severe headaches, overuse of medication, significant disability and limited access to proper headache care? What have we achieved and is it relevant for the individual headache patient? When will we find the underlying mechanisms, genetic and diagnostic markers, stratify patients and individualize their treatment? With proper animal models for both primary and secondary headaches we may understand the mechanisms and their interfaces. The pharmaceutical industry has to raise their interest in the headache and new targeted drugs should be developed. If patients at risk of chronic headache could be identified, effective prevention could be initiated in this subset of patients leading to optimization of ressources. In a world with major demographic challenges and limited ressources, education and organization in headache care are absolute essentials. These challenges and exciting possibilities will be discussed in respect to the outstanding work of Professor Jes Olesen and his generation of headache pioneers.

